# Retrospective analysis of demographic and clinical factors associated with etiology of febrile respiratory illness among US military basic trainees

**DOI:** 10.1186/s12879-014-0576-2

**Published:** 2014-12-05

**Authors:** Damaris S Padin, Dennis Faix, Stephanie Brodine, Hector Lemus, Anthony Hawksworth, Shannon Putnam, Patrick Blair

**Affiliations:** Naval Health Research Center, 140 Sylvester Road, San Diego, 92106 CA USA; Graduate School of Public Health, San Diego State University, 5500 Campanile Drive, San Diego, 92182 CA USA

**Keywords:** Adenovirus, Influenza, Military trainees, Military medicine, Diagnosis

## Abstract

**Background:**

Basic trainees in the US military have historically been vulnerable to respiratory infections. Adenovirus and influenza are the most common etiological agents responsible for febrile respiratory illness (FRI) among trainees and present with similar clinical signs and symptoms. Identifying demographic and clinical factors associated with the primary viral pathogens causing FRI epidemics among trainees will help improve differential diagnosis and allow for appropriate distribution of antiviral medications. The objective of this study was to determine what demographic and clinical factors are associated with influenza and adenovirus among military trainees.

**Methods:**

Specimens were systematically collected from military trainees meeting FRI case definition (fever ≥38.0°C with either cough or sore throat; or provider-diagnosed pneumonia) at eight basic training centers in the USA. PCR and/or cell culture testing for respiratory pathogens were performed on specimens. Interviewer-administered questionnaires collected information on patient demographic and clinical factors. Polychotomous logistic regression was employed to assess the association between these factors and FRI outcome categories: laboratory-confirmed adenovirus, influenza, or other FRI. Sensitivity, specificity, positive and negative predictive value were calculated for individual predictors and clinical combinations of predictors.

**Results:**

Among 21,570 FRI cases sampled between 2004 and 2009, 63.6% were laboratory-confirmed adenovirus cases and 6.6% were laboratory-confirmed influenza cases. Subjects were predominantly young men (86.8% men; mean age 20.8 ± 3.8 years) from Fort Jackson (18.8%), Great Lakes (17.1%), Fort Leonard Wood (16.3%), Marine Corps Recruit Depot (MCRD) San Diego (19.0%), Fort Benning (13.3%), Lackland (7.5%), MCRD Parris Island (8.7%), and Cape May (3.2%). The best multivariate predictors of adenovirus were the combination of sore throat (odds ratio [OR], 2.94; 95% confidence interval [CI], 2.66-3.25), cough (OR, 2.33; 95% CI, 2.11-2.57), and fever (OR, 2.07; 95% CI, 1.90-2.26) with a PPV of 77% (p ≤.05). A combination of cough, fever, training week 0-2 and acute onset were most predictive of influenza (PPV =38%; p ≤ .05).

**Conclusions:**

Specific demographic and clinical factors were associated with laboratory-confirmed influenza and adenovirus among military trainees. Findings from this study can guide clinicians in the diagnosis and treatment of military trainees presenting with FRI.

**Electronic supplementary material:**

The online version of this article (doi:10.1186/s12879-014-0576-2) contains supplementary material, which is available to authorized users.

## Background

Historically, military basic trainees have been vulnerable to severe epidemics of febrile respiratory illness (FRI) [1],[2]. Several factors predispose military trainees to the spread respiratory infections, including crowded living conditions and stressful work environments. The epidemiology and pathogens causing FRI among military trainees is unique and differs from the civilian setting [3]. Training populations are primarily prone to outbreaks caused by adenovirus and influenza, which often co-circulate and present with similar clinical signs and symptoms [4].

In the presence of an epidemic, it is generally acceptable to make a reasonable diagnosis based on clinical signs and symptoms until laboratory confirmation can be obtained. It is still debatable whether specific demographic and clinical symptoms can be used to distinguish among respiratory pathogens. A few studies have identified clinical predictors for influenza in civilian and military populations [5]-[9]. However, few studies have focused on other respiratory pathogens like adenovirus that have caused substantial morbidity among military trainees [10],[11].

Adenovirus infections have caused significant morbidity among US military trainees since the virus was discovered in the1950s. With interrupted training schedules, high morbidity, and the occasional death, it has proved to be costly to the US Government [12],[13]. Live oral vaccines against types 4 and 7 adenovirus were developed in the late 1960s and were successful in reducing FRI rates and adenoviral morbidity after their introduction in 1971. However, the sole vaccine manufacturer stopped production in 1996, and adenovirus returned to US recruit training centers and commands (RTCs) at pre-vaccine levels after vaccine supplies were depleted in 1999. The clinical presentation of adenovirus-associated FRI has been described as more severe than non-adenovirus-associated FRI, particularly when it is a novel strain in which there is little to no immunity [14]. Adenoviruses cases present with higher temperatures and have an increased number of cases with a cough and sore throat compared with non-adenovirus-associated FRI cases [14].

FRI infections present with a wide range of clinical features. Identifying demographic and clinical factors associated with the primary viral pathogens causing FRI epidemics among military trainees will help improve differential diagnosis, allow for appropriate distribution of antiviral medications, and ultimately improve troop readiness. The objective of this study was to determine what demographic and clinical factors are associated with influenza and adenovirus among military trainees at RTCs in an effort to aid clinical diagnosis when laboratory confirmation is not available.

## Methods

### Study population

Since 1996, the Naval Health Research Center (NHRC) has conducted ongoing laboratory-based surveillance FRI at eight basic training centers across the United States: Fort Benning Army Training Center, Georgia; Fort Jackson Army Training Center, South Carolina; Fort Leonard Wood Army Training Center, Missouri; Lackland Air Force Base, Texas; Great Lakes Naval Training Command, Illinois; Marine Corps Recruit Depots (MCRDs) San Diego, California, and Parris Island, South Carolina; and Cape May Coast Guard Training Center, New Jersey. Length of basic training varies throughout branches of military service: 10 weeks for the Army, 7 weeks Navy, 8 weeks Air Force, 12 weeks Marine Corps, and 8 weeks Coast Guard. In support of surveillance, military basic trainees from these training sites meeting case definition for FRI (fever ≥38.0°C with either cough or sore throat; or provider-diagnosed pneumonia) were recruited for the study. All subjects provided informed consent. This study was approved by the Naval Health Research Center (NHRC) Institutional Review Board (protocol NHRC.1999.0002).

### Specimen and data collection

Throat and nasal swabs and clinical data were systematically collected from consenting US military trainees meeting the case definition for FRI. Each training site except for Cape May had a trained research assistant dedicated to enrolling subjects in the study and collecting clinical specimens. Sampling of a maximum ranging from 10 to 20 recruits per site per week was performed. Subjects were systematically selected from trainees presenting to their local clinic meeting FRI case definition. Trainees were tracked throughout the entirety of their training, with the exception of MCRD San Diego where trainees left for Camp Pendleton for weeks 4 through 7 of training. Trainees awaiting the start of training were assigned to training week 0.

Questionnaires administered by research assistants (corpsmen at Cape May) collected information on patient demographic and clinical factors, including age, sex, number of days since symptoms began, number of visits to sick call during illness, diagnosis of pneumonia, history of asthma, smoking history (pack-years), history of influenza vaccination, week of training, and experience of any of the following symptoms during his or her current illness: sore throat, cough, shortness of breath, nasal congestion, headache, conjunctivitis, body aches, and nausea or vomiting. The patient's oral temperature was abstracted from the medical record or taken by the research assistant. Medical records were reviewed to abstract data needed for questionnaire.

### Specimen processing

Up to two throat and/or nasal swabs were collected by on-site personnel using Dacron respiratory swabs (MicroTest viral transport media (VTM), Remel, Inc., Lenexa, KS, USA). Swabs were immediately placed into the VTM and broken off so that the tip remained in the media. VTM were maintained on ice or refrigerated at 4°C (2-8°C) during processing and stored within 60 minutes at -70°C. Specimens were stored locally at -70°C until shipped on dry ice to NHRC for viral culture and molecular diagnostic processing. Shipping occurred on a weekly or biweekly basis (not to exceed 1 month) depending on season and FRI rates at respective training sites. The NHRC laboratory is accredited by the College of American Pathologists.

Upon arrival at NHRC, all specimens underwent molecular testing (polymerase chain reaction [PCR]) for a number of respiratory pathogens, including adenovirus and influenza A. Viral culture was performed on 20% of randomly selected specimens that were PCR positive for adenovirus and on all of the specimens that were PCR negative for adenovirus. Viral culture was performed using two different cell lines: primary monkey kidney cell line (either rhesus or cynomolgus) for influenza and an A549 cell line for adenovirus. Influenza-positive isolates were typed using hemagglutination inhibition, and adenovirus isolates were typed using microneutralization techniques [15],[16]. Viral culture was used to test for influenza A and B and other FRI pathogens (respiratory syncytial virus, and parainfluenza viruses 1 and 3).

### Data handling

The main outcome variables were laboratory-confirmed influenza or adenovirus, defined as having any specimen positive for influenza or adenovirus using PCR or culture. Adenovirus and influenza A results were determined by PCR or culture, and influenza B was determined by culture alone. The main outcome variable was categorized into a three-level variable based on FRI laboratory determination: adenovirus positive, influenza positive, or other FRI (negative for adenovirus and influenza). Categorical variables were constructed from continuous clinical variables based on clinical definitions. Acute onset was defined as ≤3 days since symptom onset. Fever was defined as temperature ≥38.0°C. Those who were afebrile were enrolled as provider-diagnosed pneumonia cases. Assuming influenza vaccine protection takes 14 days to develop, the influenza-vaccinated group was defined as those vaccinated longer than 14 days prior to testing. Data from the 2009 H1N1 pandemic were included in the analysis.

### Statistical analysis

Statistical analysis was performed using SAS software, version 9.2 (SAS Institute Inc., Cary, NC, USA). Frequencies and descriptive statistics were performed. Mean and standard deviation were reported for normally distributed variables, and median and range were reported for non-normally distributed variables. A multivariate polychotomous logistic regression model was developed to model the probability of laboratory-confirmed adenovirus, laboratory-confirmed influenza, and other FRI against clinical and demographic predictors (age, sex, acute onset, fever, pneumonia diagnosis, sore throat, cough, shortness of breath, nasal congestion, headache, conjunctivitis, body aches, nausea/vomiting, asthma, smoking history, influenza vaccination status, training week, and enrollment site). Colinearity diagnostics were done to assess confounding between predictor variables. First clinic visit and cigarette pack-years were not incorporated in the model because of the possibility of collinearity with acute onset and smoking.

Manual stepwise model-building strategy was used to develop the model that included all variables significantly associated with the main outcome variables. Predictors were included in the model one at a time and removed if not significant at p <0.05. Asthma was the only predictor that fell out of the model. Parameter estimates and significance levels were reviewed during model building to assess the relative importance of each variable. Adjusted odds ratios (ORs) and confidence intervals (CIs) were reported for each multivariate logistic regression model. Variance inflation factor and tolerance values were evaluated to assess multicollinearity and model fitness. All variables were considered statistically significant at p <0.05.

Variables demonstrating associations with laboratory-confirmed adenovirus and influenza (ie, the predictors of influenza and adenovirus) in the reduced multivariate model were incorporated into various clinical combinations. Sensitivity, specificity, positive predictive value (PPV), and negative predictive value were calculated for individual predictors and various clinical combinations of predictors in an effort to model these as clinical diagnostic tests. Binomial proportions with 95% CIs were calculated.

## Results

### Descriptive analysis

Among 21,570 FRI cases sampled between November 2004 and October 2009, 63.6% were laboratory-confirmed adenovirus cases and 6.7% were laboratory-confirmed influenza cases. Trainees presented to the clinic 3 days after self-reported onset of symptoms (range 1-90). For 68.0% of trainees, it was their first illness-related clinic visit. Subjects were predominantly young men (86.8% men; mean years 20.8 ± 3.8) from Fort Jackson (18.8%), Great Lakes (17.1%), Fort Leonard Wood (16.3%), MCRD San Diego (19.0%), Fort Benning (13.3%), Lackland (7.5%), MCRD Parris Island (8.7%), and Cape May (3.2%). Most subjects were febrile (83.0%) and had a cough (89.2%) and/or sore throat (88.9%) because of study inclusion criteria. Adenovirus-confirmed cases had a higher percentage with a sore throat compared with influenza cases (adenovirus = 93%, influenza = 81.3%; P < .0001). Conversely, influenza-positive cases had a higher percentage with a cough compared with adenovirus-positive cases (influenza = 98.3%, adenovirus = 91.5%; P < .0001). Subject characteristics are reported in Table 1.

**Table 1:** **Demographic and clinical characteristics of military trainees according to etiology of febrile respiratory illness (2004-2009)**

Variable by FRI outcome	Total study population	Adenovirus	Influenza	Other
	( ***n*** = 21,570)	( ***n*** = 13,725)	( ***n*** = 1,435)	( ***n*** = 6,410)
Continuous variables^e^	value^a^	value^a^	value^a^	value^a^
Age (years)	20.8 (3.8)	20.5 (3.7)	21.2 (3.7)	21.2 (4.2)
Symptom duration (days)	3.0 (1-90)	3.0 (1-90)	3.0 (1-52)	3.0 (1-84)
Temperature (°C)	38.2 (1.5)	38.4 (1.5)	38.4 (1.3)	38.2 (1.5)
Smoking history (pack-years)	1.0 (0-99)	1.0 (0-99)	1.0 (0-72)	0.5 (0-40)
Days since vaccination^b^ (Influenza)	31.0 (0-365)	33.0 (0-363)	10.0 (0-351)	29.0 (0-365)
Training week	4.7 (3.0)	5.0 (2.6)	3.2 (3.2)	4.7 (3.8)
Categorical variables^e^	*n* (%)	*n* (%)	*n* (%)	*n* (%)
Training season				
Fall	5,088 (23.6)	3,423 (24.9)	227 (15.8)	1,438 (22.4)
Spring	4,675 (21.7)	3,144 (22.9)	78 (5.4)	1,453 (22.7)
Summer	6,574 (30.5)	4,501 (32.8)	403 (28.1)	1,670 (26.1)
Winter	5,233 (24.3)	2,657 (19.4)	727 (50.7)	1,849 (28.8)
Sex				
Female	2,846 (13.2)	1,568 (11.4)	252 (17.6)	1,026 (16.0)
Male	18,724 (86.8)	12,157 (88.6)	1,183 (82.4)	5,384 (84.0)
Acute onset				
≤3 days	12,750 (59.1)	7,623 (55.5)	1,060 (73.9)	4,067 (63.4)
>3 days	8,820 (40.9)	6,102 (44.5)	375 (26.1)	2,343 (36.6)
Fever				
≥38.0°C	17,917 (83.0)	11,945 (87.0)	1,232 (85.9)	4,740 (73.9)
<38.0°C	3,653 (17.0)	1,780 (13.0)	203 (14.1)	1,670 (26.1)
First visit				
Yes	14,659 (68.0)	8,867 (64.6)	1,065 (74.2)	4,727 (73.7)
No	6,911 (32.0)	4,858 (35.4)	370 (25.8)	1,683 (26.2)
Pneumonia^c^				
Yes	2,882 (13.4)	1,624 (11.8)	107 (7.5)	1,151 (18.0)
No	18,688 (86.6)	12,101 (88.1)	1,328 (92.5)	5,259 (82.0)
Sore throat				
Yes	19,174 (88.9)	12,771 (93.0)	1,168 (81.3)	5,235 (81.7)
No	2,396 (11.1)	954 (7.0)	267 (18.6)	1,175 (18.3)
Cough				
Yes	19,249 (89.2)	12,559 (91.5)	1,410 (98.3)	5,280 (82.4)
No	2,321 (10.7)	1,166 (8.5)	25 (1.7)	1,130 (17.6)
Shortness of breath				
Yes	8,045 (37.3)	5,010 (36.5)	545 (38.0)	2,490 (38.8)
No	13,525 (62.7)	8,715 (63.5)	890 (62.0)	3,920 (61.2)
Nasal congestion				
Yes	17242 (80.0)	11223 (81.8)	1153 (80.3)	4,866 (75.9)
No	4328 (20.1)	2502 (18.2)	282 (19.7)	1,544 (24.1)
Headache				
Yes	17,890 (82.9)	1,1571 (84.3)	1,204 (84.0)	5,115 (79.8)
No	3680 (17.1)	2154 (15.7)	231 (16.0)	1,295 (20.2)
Conjunctivitis				
Yes	1,901 (8.8)	1,224 (8.9)	76 (5.3)	601 (9.4)
No	19,669 (91.2)	12,501 (91.1)	1,359 (94.7)	5,809 (90.6)
Body aches				
Yes	15,318 (71.0)	9,804 (71.4)	1,073 (74.8)	4,441 (69.3)
No	6,252 (29.0)	3,921 (28.6)	362 (25.2)	1,969 (30.7)
Nausea/vomiting				
Yes	10,757 (49.9)	7,135 (51.9)	701 (48.9)	2,921 (45.6)
No	10,813 (50.1)	6,590 (48.1)	734 (51.1)	3,489 (54.4)
Asthma				
Yes	1,153 (5.3)	670 (4.9)	85 (5.9)	398 (6.2)
No	20,417 (94.7)	13,055 (95.1)	1,350 (94.1)	6,012 (93.8)
Smoker				
Yes	6,942 (32.2)	4,360 (31.8)	406 (28.3)	2,176 (33.9)
No	14,628 (67.8)	9,365 (68.2)	1,029 (71.7)	4,234 (66.1)
Vaccinated (influenza)^d^				
Yes	14,005 (65.0)	8,685 (63.3)	888 (61.9)	4,432 (69.1)
No	7,565 (35.0)	5,040 (36.7)	547 (38.1)	1,978 (30.9)

^a^Continuous variable values reported as “mean (standard deviation)” if symmetric variable or “median (min–max range)” if non-symmetric.

^b^Missing data due to seasonal vaccine schedule-Total population, *n* = 6,067; Adenovirus group, *n* = 3,670; Influenza group, *n* = 695; Other group, *n* = 1,702. Vaccination days were limited to vaccination within the last year.

^c^All pneumonias were provider diagnosed.

^d^Individuals were considered vaccinated if their vaccination date was ≥14 days.

^e^p-value determination - χ^2^ was used for categorical variables; Kruskal Wallis test or one-way ANOVA for continuous variables. All variable were significant at P < .0001 except for shortness of breath (P =0.0300), body aches (P = 0.002), and asthma (P = 0.0002).

### Multivariate analysis

Regression modeling revealed associations with laboratory-confirmed adenovirus and clinical predictors (adjusted OR and 95% CI reported): sore throat (OR, 2.94; 95% CI, 2.66-3.25, cough (OR, 2.33; 95% CI, 2.11-2.57), fever (OR, 2.07; 95% CI, 1.90-2.26), nausea/vomiting (OR, 1.19; 95% CI, 1.11-1.27), and nasal congestion (OR, 1.12; 95% CI, 1.03-1.22). Regression modeling of laboratory-confirmed influenza showed significant associations with cough (OR, 13.85; 95% CI, 9.14-20.97), fever (OR, 1.99; 95% CI, 1.66-2.38), acute onset (OR, 1.46; 95% CI, 1.26-1.68), and body aches (OR, 1.31; 95% CI, 1.13-1.53). Sore throat, nasal congestion, and nausea/vomiting were associated with laboratory-confirmed adenovirus but not with influenza, while acute onset of illness (≤3 days) and body aches were associated with laboratory-confirmed influenza but not with adenovirus. Fever and cough were significantly associated with both adenovirus and influenza. Asthma fell out of the model all together.

**Figure 1:**
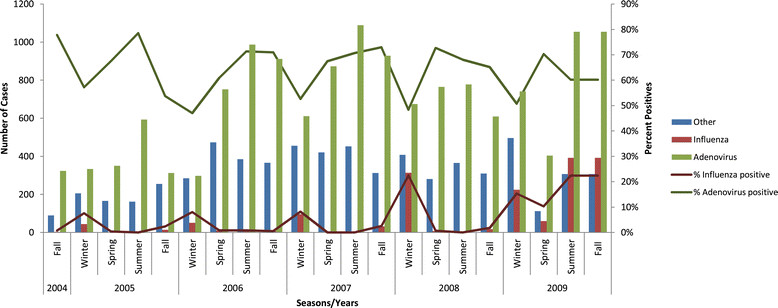
**Distribution of laboratory-confirmed influenza and adenovirus cases among military basic trainees, 2004-2009.** Histogram depicts all laboratory-confirmed influenza and adenovirus cases in the study population, along with those that were negative for influenza and adenovirus (“other” category), by season and year. Percentage of subjects who were influenza positive and adenovirus positive is also depicted.

Analysis of demographic factors revealed interesting results. Female sex was a protective factor for adenovirus infection (OR, 0.72; 95% CI, 0.66-0.80) but was not associated with influenza. Being a smoker before training was protective for both adenovirus and influenza (OR, 0.92; 95% CI, 0.86-0.99) and OR, 0.77; 95% CI, 0.67-0.88, respectively). Adenovirus was seen year-round and associated with all training seasons, with a protective effect seen during the winter season. However, winter training was significantly associated with influenza, with a protective effect seen during other seasons. Distribution of study cases by seasons and years is illustrated in the Figure 1.

**Figure 2:**
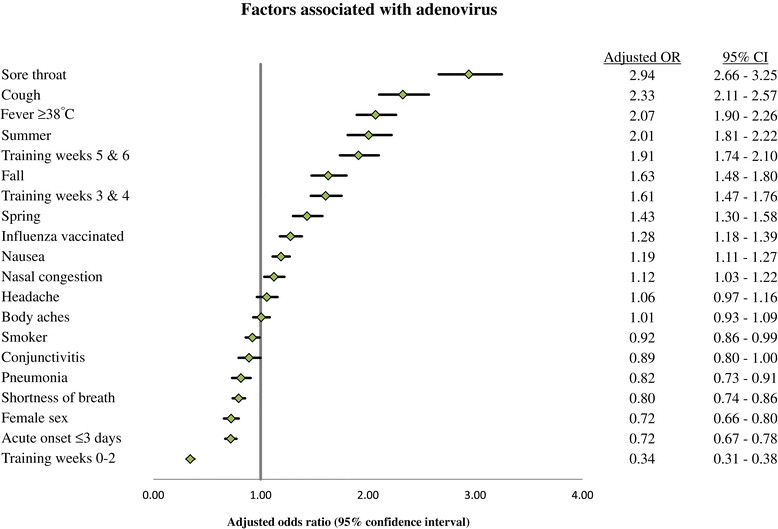
**Results of multivariate analysis comparing demographic and clinical factors of military trainees with illness due to adenovirus.** Training week was converted to a categorical variable for multivariate analysis (reference category = weeks 7+). Winter was used as the reference category for training season.

**Figure 3:**
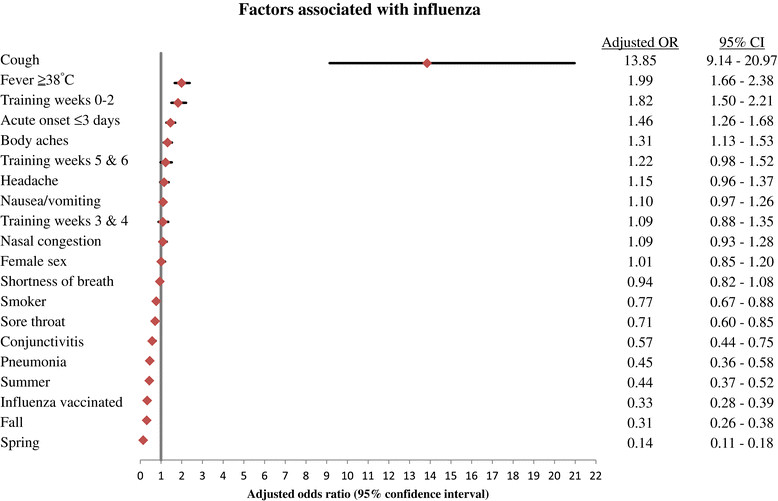
**Results of multivariate analysis comparing demographic and clinical factors of military trainees with illness due to influenza.** Training week was converted to a categorical variable for multivariate analysis (reference category = weeks 7+). Winter was used as the reference category for training season.

Furthermore, influenza was associated with the first 2 weeks of training (OR, 1.82, 95% CI, 1.50-2.21). Adenovirus was associated with weeks 3 and 4 of training (OR, 1.61; 95% CI, 1.47-1.76), these odds increased during training weeks 5 and 6 (OR, 1.91; 95% CI, 1.74-2.10), with no association seen with influenza during training weeks 3-6. Clinical and demographic factors associated with the development of laboratory-confirmed adenovirus and influenza, as determined by multivariate polychotomous logistic regression analysis, are shown in Figures 2 and 3.

Table 2 displays the performance of clinical diagnostic tests constructed from combinations of predictors for adenovirus. The highest specificity (92.4%) and PPV (81.7%) were noted for a combination of four criteria (sore throat, fever, training weeks 3-6, summer season), but the sensitivity was low (19.5%). The combination of three criteria (sore throat, cough, and fever) yielded both high sensitivity (74.4%) and PPV (71.2%) but had marginal specificity (47.3%). Table 3 displays the results of clinical diagnostic tests constructed from combinations of predictors for influenza. The combination of four criteria (cough, training week 0-2, winter season, and fever) had the highest specificity (97.0%) and PPV (39.4%) but had a sensitivity of 26.9%. The combination of two criteria (cough and fever) showed the highest sensitivity (84.3%), with a specificity of 27.1% and a PPV of 7.6%.

**Table 2:** **Clinical diagnostic tests based on main predictor variables associated with adenovirus among military trainees with febrile respiratory illness**

Clinical predictor(s)	SN (%) (95% CI)	SP (%) (95% CI)	PPV (%) (95% CI)	NPV (%) (95% CI)
Sore throat	93.0 (92-93)	18.4 (17-19)	66.6 (65-67)	60.2 (58-62)
Cough	91.5 (91-91)	14.7 (13-15)	65.2 (64-65)	49.8 (47-51)
Fever	87.0 (86-87)	23.9 (22-24)	66.7 (65-67)	51.3 (49-52)
Summer	32.8 (32-33)	73.4 (72-74)	68.5 (67-69)	38.3 (37-39)
Training week 5 & 6	30.2 (29-30)	83.5 (82-84)	76.2 (75-77)	40.6 (39-41)
Fall	24.9 (24-25)	78.8 (77-79)	67.3 (65-68)	37.5 (36-38)
Training week 3 & 4	38.8 (38-39)	76.9 (75-77)	74.6 (73-75)	41.8 (41-42)
Training week 3-6	70.0 (69-70)	59.4 (58-60)	75.3 (74-76)	52.9 (51-53)
Spring	22.9 (22-23)	80.5 (79-81)	67.3 (65-68)	37.4 (36-38)
Sore throat + cough	85.3 (84-85)	30.6 (29-31)	68.3 (67-68)	54.4 (52-55)
Sore throat + fever	81.2 (80-81)	36.7 (35-37)	69.2 (68-69)	52.7 (51-54)
Sore throat + cough + fever	74.4 (73-75)	47.3 (46-48)	71.2 (70-71)	51.4 (50-52)
Sore throat + cough + fever + summer	24.2 (23-24)	86.3 (85-87)	75.5 (74-76)	39.4 (38-40)
Sore throat + cough + fever + training week 3-6	52.8 (51-53)	79.0 (78-79)	81.5 (80-82)	48.9 (48-49)
Sore throat + fever + summer	26.7 (25-27)	83.0 (82-83)	73.4 (72-74)	39.3 (38-40)
Sore throat + fever + training week 3-6	56.7 (55-57)	75.4 (74-76)	80.1 (79-80)	49.9 (48-50)
Sore throat + fever + training week 3-6 + summer	19.5 (18-20)	92.4 (91-92)	81.7 (80-82)	39.6 (38-40)

NOTE. Predictor variables were added to diagnostic test based on descending order of significance with outcome variable (influenza) on multivariate logistic regression results. Adenovirus was compared with FRI other (non-adenovirus confirmed, non-influenza confirmed) and influenza confirmed.

CI, confidence interval, calculated with binomial expansion; NPV, negative predictive value; PPV, positive predictive value; SN, sensitivity; SP, specificity.

**Table 3:** **Clinical diagnostic tests based on main predictor variables associated with influenza among military trainees with febrile respiratory illness**

Clinical predictor(s)	SN (%) (95% CI)	SP (%) (95% CI)	PPV (%) (95% CI)	NPV (%) (95% CI)
Cough	98.3 (97-98)	11.4 (10-11)	7.3 (6-7)	98.9 (98-99)
Fever	85.9 (83-87)	17.1 (16-17)	6.9 (6-7)	94.4 (93-95)
Training week 0-2	54.0 (51-56)	84.0 (83-84)	19.4 (18-20)	96.2 (95-96)
Acute onset	73.9 (71-76)	41.9 (41-42)	8.3 (7-8)	95.7 (95-96)
Body aches	74.8 (72-77)	29.3 (28-29)	7.0 (6-7)	94.2 (93-94)
Winter	50.7 (48-53)	77.6 (77-78)	13.9 (12-14)	95.7 (95-95)
Cough + fever	84.3 (82-86)	27.1 (26-27)	7.6 (7-8)	96.0 (95-96)
Cough + training week 0-2	53.4 (50-56)	85.9 (85-86)	21.3 (19-22)	96.3 (96-96)
Cough + training week 0-2 + fever	43.7 (41-46)	89.4 (88-89)	22.7 (21-24)	95.7 (95-95)
Cough + training week 0-2 + fever + acute onset	33.2 (30-35)	93.9 (93-94)	27.9 (25-30)	95.2 (94-95)
Training week 0-2 + winter	9.3 (7-10)	95.4 (95-95)	12.6 (10-14)	93.7 (93-93)
Cough + training week 0-2 + winter	33.9 (31-36)	95.8 (95-96)	36.3 (33-38)	95.3 (95-95)
Cough + training week 0-2 + winter + fever	26.9 (24-29)	97.0 (96-97)	39.4 (36-42)	94.9 (94-95)

NOTE. Predictor variables were added to diagnostic test based on descending order of significance with outcome variable (influenza) on multivariate logistic regression results. Influenza was compared with other FRI (non-adenovirus confirmed, non-influenza confirmed) and adenovirus confirmed.

CI, confidence interval, calculated with binomial expansion; NPV, negative predictive value; PPV, positive predictive value; SN, sensitivity; SP, specificity.

## Discussion

Adenovirus and influenza infections are the leading cause of FRI outbreaks among military trainees at RTCs. This study compared the predictive values of demographic and clinical factors for laboratory-confirmed adenovirus and laboratory-confirmed influenza from 2004 to 2009 among military trainees. The results of this study may help clinicians distinguish an influenza or adenovirus infection from an illness caused by other FRI, based on clinical symptoms and demographic factors. Distinguishing clinical and demographic factors may aid clinicians with differential diagnosis when laboratory confirmation is not available and allow for appropriate dissemination of antiviral medications for influenza.

Few studies have examined factors associated with specific viral pathogens causing FRI among military populations. A study by McNeill and colleagues investigated the clinical presentation of influenza, adenovirus, and other pathogens causing FRI among military members. They found that overall viral pathogens presented as clinically indistinguishable from one another [17]. Conversely, this present study found several clinical symptoms to be independently associated with adenovirus (sore throat, nausea/vomiting, and nasal congestion) and influenza (acute onset and body aches). Cough and fever were associated with both adenovirus and influenza. However, although cough and fever were associated with both pathogens, recruits with a cough were significantly more likely to be positive for influenza (OR, 13.6; 95% CI, 9.03-20.48) than adenovirus (OR, 2.32; 95% CI, 2.07-2.59). This is consistent with other studies that have reported fever and cough as highly predictive of influenza infections, and with the Centers for Disease Control and Prevention definition for influenza-like illness [6],[18],[19].

The clinical presentation of adenovirus illness described in this paper is similar to other military studies [17],[20]. A study conducted by Brosch et al. looking at clinical predictors for adenovirus 14 showed that the majority of adenovirus cases had cough and fever [18]. McNeill et al. found similar results [17],[20]. Several other studies have seen an association between adenovirus infection and either abdominal pain or nausea [5],[17],[21], consistent with the present study. A recent study evaluating the clinical presentation of military personnel presenting with FRI found that influenza-positive cases were more likely to present with a runny nose, chills, and ocular symptoms than were other FRI cases, which were more likely to present with a sore throat and nausea/vomiting [8]. Interestingly, our study found sore throat and nausea/vomiting to be solely associated with adenovirus.

In this study, demographic factors associated with adenoviral infections included male sex, smoking, training week (weeks 5 and 6) and training season (spring, summer, and fall months). The protective association seen between smoking and both adenovirus and influenza infections appears counterintuitive. Although the literature is conflicting, a protective effect has previously been seen with adenovirus infections [22]. However, the majority of studies published have shown smoking is a risk factor for FRI [21],[23],[24]. That said, all military training facilities are smoke free and the protective effect seen among adenovirus-positive individuals may be a result of more frequent adenovirus infections seen among smokers in general, hence a natural immunity may have been present prior to military enlistment [25]. Our results substantiate previous studies that reported adenovirus-associated illness in training weeks 3-6 and the protective effect of female sex [11],[12],[26],[27]. We found that influenza was more likely to occur within the first 2 weeks of training and during the winter season, whereas adenovirus was more likely to occur during weeks 3-6 of training and during the spring, summer, and fall seasons. Training week and seasons are potential tools that clinicians can use to aid in differential diagnosis of FRI among military trainees. Clinical predictors found in this study can aid with quarantine procedures during and FRI outbreak thus reducing the spread of disease and reducing costs to the US government.

## Conclusions

After a 12-year absence, second-generation oral vaccines for adenovirus types 4 and 7 resumed distribution at RTCs in October 2011. Since that time, rates of FRI and adenoviral morbidity have decreased substantially. Although there is hope that the cross-protective effect of the vaccines will be seen among the non-vaccine-targeted adenovirus serotypes, it will be important to track the response of different serotypes to the vaccines. The body of literature could be enhanced by conducting further studies examining clinical and demographic characteristics of adenovirus subtypes causing FRI at RTCs, with a comparative analysis pre- and post-vaccine distribution.

Although our study included a robust sample size and laboratory-confirmed illness, it predominately consisted of young men in a military training environment. The epidemiology and pathogens causing respiratory illness among military recruits is often unique and different from that of the civilian setting. As a result, these finding may not be generalizable to the civilian population. Lastly, clinical predictors of influenza and adenovirus may differ among viral subtypes. Further studies are needed to explore the clinical presentation among sub-types of viral pathogens.

## Authors' contributions

DSP participated in the design of the study, coordinated study activities, conducted data analysis, and drafted the manuscript. DF conceived of the study and participated in its design, supervised laboratory reference testing, and participated in manuscript revision. SB participated in the design of the study and manuscript revision. HL participated in the design of the study and assisted with data analysis. AH and SP assisted with data analysis, participated in the design, and assisted in manuscript revision. PB participated in manuscript revision. All authors read and approved the final manuscript.

## Authors' information

Ms. Padin is a study coordinator and epidemiologist at the Naval Health Research Center (NHRC). Dr. Brodine is a researcher at NHRC, and a professor and Epidemiology Division Head at San Diego State University (SDSU) Graduate School of Public Health (GSPH). Dr. Lemus is an assistant professor at SDSU GSPH. Dr. Blair is department head for the NHRC Operational Infectious Diseases (OID) Department. Dr. Faix is assistant department head for NHRC OID. Mr. Hawksworth is the senior data manager and surveillance study coordinator for NHRC OID. Dr. Putnam is head of the Enteric Disease Surveillance Program for NHRC OID.

## Authors’ original submitted files for images

Below are the links to the authors’ original submitted files for images. Authors’ original file for figure 1 Authors’ original file for figure 2 Authors’ original file for figure 3
