# Supplementary figures and images for: Retrospective analysis of demographic and clinical factors associated with etiology of febrile respiratory illness among US military basic trainees
Source: BMC Infect Dis. 2014 Dec 5;14:576. doi: 10.1186/s12879-014-0576-2 (PMC4264259; doi:10.1186/s12879-014-0576-2)

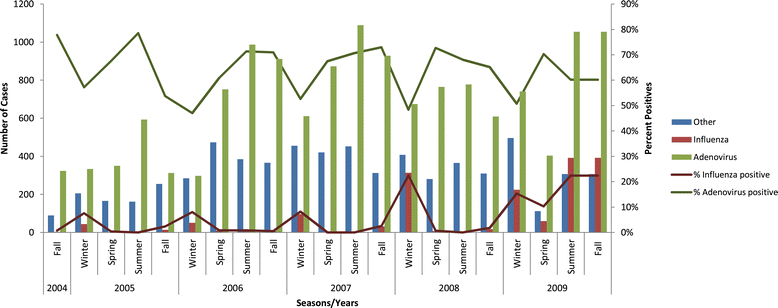

Supplement: Supplementary file 1 — Authors’ original file for figure 1 [file 12879_2014_576_MOESM1_ESM.gif]

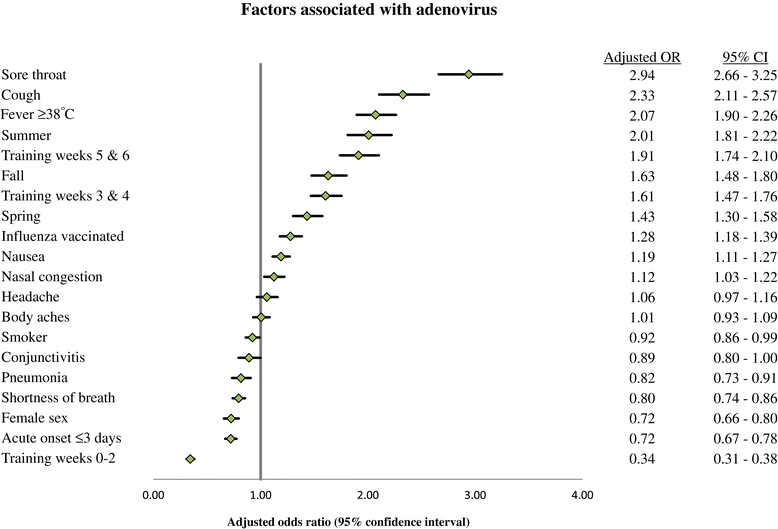

Supplement: Supplementary file 2 — Authors’ original file for figure 2 [file 12879_2014_576_MOESM2_ESM.gif]

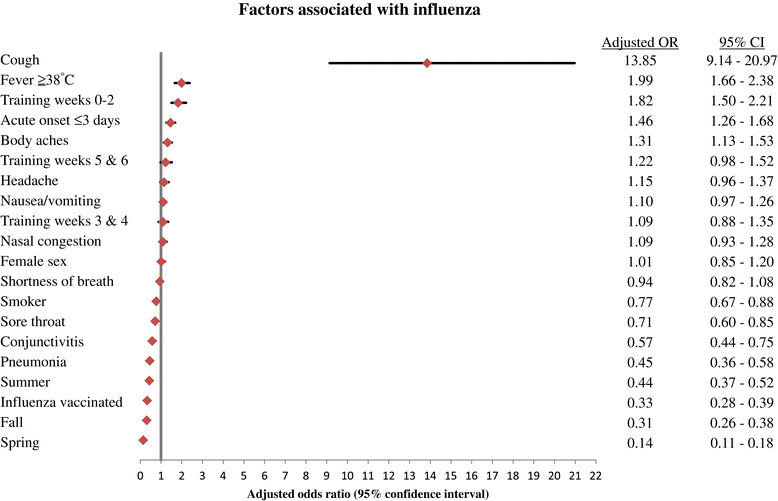

Supplement: Supplementary file 3 — Authors’ original file for figure 3 [file 12879_2014_576_MOESM3_ESM.gif]
